# The Hospital Nursing Supplement

**Published:** 1894-06-23

**Authors:** 


"The Hospital" Nursing Mirror.

Being the Extra Nursing Supplement of "The Hospital" Newspaper.

[Contributions for this Supplement should be addressed to the Editor, The Hospital, 428, Strand, London, W.C., and should have the word "Nursing" plainly written in left-hand top corner of the envelope.]

### News from the Nursing World.

### OUR PRINCESS.

Her Royal Highness the Princess of Wales has kindly consented to receive personally donations and collections of five pounds and upwards on the occasion of the opening of the new buildings of the British Home for Incurables at Streatham on July 2nd. Special sachets and collecting cards can be obtained from the Secretary. Seats will be reserved for those making the presentations. A Country Fair and Market will be held in the grounds on July 2nd, 3rd, and 4th, when a most varied and charming programme of amusements will be carried out.

### THE NURSES' CO-OPERATION.

A general meeting of the nurses of the Co-operation was held at 8, New Cavendish Street, on June 13th, a large number of members being present. An interesting discussion followed several good speeches, and it was proposed and seconded by the nurses, and carried by a large majority of votes, that the distribution of a bonus should not take place at present, or before the end of five years from the foundation of the society. The extremely prosperous condition of the Co-operation would justify an immediate distribution, but we congratulate them on their decision to allow the surplus funds to remain intact for another two years. The members of the Nurses' Co-operation, 8, New Cavendish Street, may certainly pride themselves upon its sound financial position, and so also may the Lady Superintendent, to whose indefatigable exertions the movement owes so much of its success. An "At Home" for nurses belonging to the Co-operation has been arranged to take place on the first Friday in each month, from four to seven, at the offices of the association.

### TRAINED AND UNTRAINED.

Two ladies were going over a small hospital nursed by worthy but untrained sisters. "It is all most beautiful," remarked one of the visitors enthusiastically; "so clean, and the patients must love to be waited on by such gentle creatures." The other lady remained silent until the street was reached. "You saw the man who was so bad," she remarked then to her friend, "well, he has acute rheumatism, and he was wearing only a cotton shirt, and that was too small, and the shoulder and arm, where, he said, the chief pain was centred, were quite close to that large, ill-fitting window." The enthusiastic lady looked depressed. "But the wards were surely delightfully clean?" she protested. "Yes," said the more observant visitor, "and bare, too; there was no occupation or amusement whatever for the patients lying there thinking of their troubles. Suitable diversion is as needful as proper medicine." "But surely they are well nursed," said the lady, anxious to retain one, at any rate, of her first impressions. But her experienced friend answered, "Not in the true sense of the word. They are looked after kindly by day, but they are left alone at night, and occasionally a typhoid case *does* get a little solid food from his neighbour, which is grievous but unavoidable under the present system."

### COMPLAINTS DISCOURAGED.

"Have you any complaints to make?" the Visiting Committee asks of the patients or nurses at stated intervals. Knowing by experience that a reputation for grumbling is the most damaging possession possible for a resident in any institution, "No thank you, sir," is the usual reply. "Food good?" "Pretty fair, sir," and the visitors go away feeling virtuous and complacent. They are well fed and independent gentlemen, who never realise that there is any moral restraint on free speech in the place. Of course it never occurs to them that if a nurse says the milk is not good and the potatoes bad, she is aware that she renders herself liable to be summoned by the steward or other official, and constrained to prove or else retract her honest criticism. He naturally will try to shift the blame by asserting that her "milk cans could not have been clean" or she must have let the potatoes "stand too long." In spite of her convictions to the contrary she is probably driven to own that this may be the case. Thus her attempted straightforward act in the patients' behalf is so effectually checked that the results of her courage are *nil* as regards them, whilst it has caused her to be regarded with suspicion by every official in the place.

### HELP URGENTLY NEEDED.

For the last seven years excellent work has been unostentatiously done at a little convalescent home at Banstead, in Surrey, called "Woodman's Cottage," for boys from four to twelve years of age. Surgical cases do especially well there, for a lengthened stay in the fine air of the Epsom Downs sometimes obviates the necessity for an operation, whilst it also builds up the strength of those for whom active surgical treatment is required. The little home has proved too of great value to many little convalescents after they are discharged from hospital, and the charge for each patient is only 5s. per week, with 6d. for washing. The small house which was temporarily adapted for the home has now, from a variety of causes, become unsuitable for the purpose, and must be replaced by a new building. If this is not done the good work will be hindered, if not altogether arrested, a prospect which no one concerned will be willing to permit. To secure renewed health to a succession of delicate children is surely a noble ambition, and only £700 is needed for the purchase of a site and erection of suitable premises. The generous public, to whom the pains and weakness of little children seldom appeal in vain, may surely be trusted to take up this matter. All particulars can be obtained from Miss Clarke, Woodman's Cottage, Banstead, Surrey, to whom also subscriptions may be sent.

### AN INQUIRY COURTED.

The nursing arrangements at Bath Workhouse are somewhat widely discussed just now, and, therefore, they will doubtless eventually be amended. Much light has been recently thrown upon the internal management by correspondence appearing in the local press. It is to be hoped that the appeal of the medical officer for a Local Government Board inquiry will be promptly complied with, for Dr. Craddock appears to have championed the cause of the helpless sick so bravely that his recommendations deserve every consideration. He names amongst other cases that of a pauper who died of typhoid, and who during his illness was entirely left in the charge of the pauper inmates by night and day. For the sick children old women of 69 and 75 seem to have proved inefficient caretakers. All honour should be shown to those medical officers, trained nurses, and others who courageously espouse the cause of the poor, often it must be owned, in the face of most discouraging opposition.

### PET ECONOMIES.

Everybody has a special "thrifty" point, and some people have more than one cherished economy. Each mentally notes the "close" or "mean" way of another, thinking it less admirable than his own. Economies practised merely at the expense of personal discomfort are of course generally considered less objectionable than those which bring inconvenience on other people. The ingenious methods by which guardians sometimes defend misplaced economy, protesting that "they are dealing with ratepayers' money," is chiefly observable when a subscription is asked for some local nursing association. They own cheerfully their belief that nurses have done much for patients who would otherwise have burdened the rates, and have attended dozens of persons free of charge. Yet these facts are not always looked upon as arguments in favour of helping the fund which supplies them. On the contrary, it is but rarely that we hear of a donation ungrudgingly given. The plea is sometimes put forth that if a request for a subscription were acceded to, some other kindred association might ask for aid. So economy is practised regardless of justice or of the wishes of the ratepayers themselves. The latter would surely think a few pounds well invested in securing skilled nursing at home for sick paupers.

### AFTER FIVE YEARS.

After having worked for five years as a nurse in Chesterfield Hospital, Miss Cole has been appointed by the Guardians to the post of nurse at the female hospital. As reported in the local press, she is to receive 9s. a week in lieu of rations, and a salary of £20 per annum. If Miss Cole has done so well during her five years' service that the Guardians feel they have consulted the best interests of the sick poor in engaging her again, it seems unfortunate that they can only give such low pay for skilled labour. It is a mistake to make a hospital nurse "keep herself." For hard-working women it is obviously desirable that suitable meals should be regularly provided, and leisure secured for their proper digestion.

### PRINCE ALFRED HOSPITAL, SYDNEY.

The annual report of this beautiful hospital, recently issued, proves a most satisfactory one. The work of the institution grows steadily and the best results possible are obtained in it. After courteously and gratefully acknowledging their "deep sense of gratitude to the honorary medical and surgical staff of the hospital, for the ability, zeal, and kindness they have shown in their attendance upon the patients during the year," the directors cordially testify to the ability, diligence, and enthusiasm of the resident staff, and the Matron (Miss McGabey), who has devoted herself with much energy, self-sacrifice, and ability to the numerous duties of her department, to the promotion of efficiency in the nursing staff, and of the material and social well-being of the nurses. During the year evening lectures have been given to the nurses by Dr. Hinder, Dr. Jenkins, Dr. McAllister, Dr. Purser, Dr. Chisholm Ross, and Professor Wilson on anatomy, physiology, surgical and medical nursing, materia medica, the nursing of insane, delirious, and nervous patients, gynæology, electricity, and massage. Lectures on elementary nursing have been given by the Matron, and invalid cookery taught by Miss Keegan. The probationers at Prince Alfred Hospital are decidedly to be congratulated upon the thoroughness of the training provided for them, and the high reputation of the present nursing staff reflects great credit on those who have organised this admirable school.

### ANOTHER BRAVE NURSE.

The lady who is now head of the Babies' Hospital in New York was recently the victim of a serious accident, the results of which necessitated her returning as a patient to the New York Hospital, where she was originally trained. Some clothing in the children's ward was set alight by a spirit lamp, and Miss Wheeler by her courage and promptitude succeeded in saving the life of a nurse and rescuing the children from imminent danger, incurring severe burns herself whilst doing so. It is not surprising to learn that this brave nurse was looked upon as a heroine when she was carried to her old ward in the new capacity of patient.

### SHORT ITEMS.

The Ballymena Guardians have decided to advertise for a trained nurse for the workhouse.—On 11th inst. a marriage took place between Mr. Daniel Arnott, of Pontypridd, and Miss Ida Williams, a trained nurse who has done voluntary work amongst the sick poor of Cardiff for some years.—The three nurses chosen by the Smethwick District Nursing Association commenced work on April 2nd, and had paid 1,671 visits up to May 31st.—A large number of nurses have attended the Water Show at Earl's Court, by special invitation from the managers.—The annual meeting of the Congleton Nurse Society was recently held at the Town Hall, when the association was shown to be in a thoroughly satisfactory position, the nurses' visits being increasingly appreciated.—Miss Lucy Goodacre has been appointed nurse at the Pasfield Union Hospital at a salary of £35 per annum.—On Friday, the 15th, the first of a course of lectures to the nurses was delivered at the National Hospital for Diseases of the Heart and Paralysis, in Soho Square, by Dr. George Cohen, the Resident Medical Officer.

### On General Nursing.

By Rowland Humphreys, M.R.C.S., L.R.C.P.Lond.

### XVII.—LATENT AND CHRONIC RHEUMATISM.

One form of rheumatism is the latent, in which the pains are slight. It usually occurs in children, and little notice is taken of so-called "growing pains." They pass off in time, but leave behind them too often a weak heart. Small fibrous nodules appear on the knees, ribs, or elbows during the course of the attack, and these are characteristic of rheumatism.

Another form is the chronic, the disease running a mild course, attack after attack occurring with little interval between, and bearing a great resemblance to rheumatic gout.

Then there is gonorrhœal rheumatism, due to a septic condition, in which the joints are attacked in somewhat the same way as in acute rheumatism. In a bad case the author of this paper had under his care some years ago, he treated the case as one of joint disease, putting tension on the affected limb (the knee), so that the surfaces of the joint were kept asunder, and the patient made a good recovery, and did not remain crippled as often occurs in this form of the complaint. Muscular rheumatism is another form of the disease, and is due to wet or chill. Lumbago, stiff neck, and similar complaints come into this category. Lumbago, &c., may be at once relieved in many cases by an injection of cocaine deep into the affected muscles. Salycilate of soda will also relieve it greatly.

Rheumatic people suffer from a variety of lesser ailments, such as tooth-ache and indigestion (which often precedes an attack of the acute form). These will frequently yield at once to the salycilate, where other remedies have failed.

It should also be mentioned that rheumatic fever, or a complaint having identical symptoms with it, often occurs in the course of an attack of scarlatina, and with it go the same dangers of cardiac disease.

If the case goes on well the patient will be apparently recovered in ten or fourteen days under the salycilic treatment, but there is still a great tendency to relapse, and, if the patient be permitted up, or does anything injudicious, or leaves off his medicine, often in spite of every precaution he relapses. The disease then runs its course again, and the patient is subject to the same dangers as in the first attack. The relapses may recur several times, so that the patient may spend weeks and weeks in bed. The complaint usually runs its course in some six weeks, and the patient then recovers slowly, with a weakened heart and a tendency to fresh attacks of the disease.

The subacute form of the disease runs a similar, but much milder, course altogether. The heart frequently escapes, and the tendency to relapses seems less. The patient often is about again in three weeks.

The treatment of rheumatic fever is divided into three parts: (1) the relief of the pain and discomfort; (2) the nursing, which is especially difficult from the inflammation of the joints, which renders the least movement very painful; and (3) the prevention and treatment of the heart complications. It is said that if we could administer the salycilate before the onset of cardiac inflammation, we should rarely, if ever get it; but, unfortunately, it usually occurs so early in the complaint, that it is present when the patient is first seen. The treatment by salycilic acid, either combined with soda or with some other body, is now the universal one, except where it is, from one cause or other, unsuitable. The natural compound salycin is made from willow trees, whereas the preparation generally used is made from coal tar, and often contains other bodies which cause many of the symptoms attributed to the salycilate. Thus it depresses greatly, and therefore is not employed where the heart is affected. It also causes deafness and noises in the head, and from these causes may have to be left off. It is, undoubtedly, a very valuable drug, and if given in sufficient doses will remove all pain within some forty-eight hours. Where the heart is affected another alkali is given instead, such as bicarbonate of soda. The use of an alkali is supposed to lie in the neutralisation of some acid which is circulating in the blood. The sweat, &c., in these cases is highly acid, and this acidity is supposed to be neutralized by the administration of the alkali by the mouth. It has been found that while lactic acid was being administered, as in cases of diabetes, that the patients not uncommonly got rheumatic fever, and that as soon as it was left off the rheumatism went, returning when the drug was again given. So that there is some foundation for the treatment.

While the salycilate of soda is obtaining its effect, there is plenty of opportunity for nursing, and where the drug cannot be given for one reason or another, as is not commonly the case, there is still greater necessity for the trained nursing. The pain may be relieved by hot fomentations, bearing in mind that the rheumatic joint is much more sensitive to heat than to cold. Irrigating tubes, with cold or hot water, or lotions—for example, lead and opium—sometimes relieve much, so do drugs which numb the skin. The main thing is to keep the joint bent in an easy position and fixed, the jumping of the limb being one of the principal causes of pain. It may be treated as a surgical case with considerable relief to the patient; and the nurse is then able to manage the necessary movements more easily, and with less discomfort to the patient; but this is, of course, not applicable to those cases where many joints are affected, or, at any rate, not so easily. The smaller joints may be "put up" on pasteboard which has been moulded to their shape after softening by pouring boiling water over it. The larger ones may be fixed on splints, made of wood or perforated zinc, and slung. If the nurse is single-handed or the patient very heavy, or very bad, much assistance is afforded by the use of a bed lift, the simpler the better, of which there are a number in the market, or one could readily be made by a carpenter. A carpenter is a most useful adjunct to a sick room. Besides the above instance, another case in which he is very useful, is when it is necessary to put a tent up round a lung case. Of course he would only be long enough in the room to do the fixing up of the required apparatus, doing the rough work at his bench, and the patient may be surrounded by a screen while he is at work. The patient should be clothed in flannel in such a manner that the garment can be taken off with the least possible disturbance to the patient.

The diet in acute rheumatism should consist principally of milk. This should be kept up for at least a week after all acute symptoms have gone; it is then increased slowly and gradually. If too rapidly changed, there is a considerable danger of a relapse.

Afterwards during convalescence, iron is greatly needed, and frequently cod-liver oil is of great service.

In the chronic forms of the disease specific treatment is of little use, general treatment with nourishing food, as much fresh air as possible, &c., are of far more service.

### Presentation.

On leaving Kendal Hospital, where she held the post of Matron for four years, Miss Brown, who is proceeding to Canada, was presented with a purse containing £20, and an illuminated address expressing cordial good wishes for her future.

### The Mechanism of Movement.

IV.

If we compare a dry dead bone with a fresh one from a butcher, we shall see that there is a great difference in the appearance and feel of the two. The dead bone is harsh, rough, and dry to the touch, and also looks brown and unnatural, whilst the fresh bone has a soft, slightly moist feel, like satin, and has a blue-white shining colour. The real difference between the two is also very great. The life of the bone is entirely due to the satin-like covering surrounding it, called the *Periosteum*. It is extremely tough, but so thin and delicate that it is a most difficult matter to remove it from the bone. This membrane contains the minute blood vessels which nourish the bone and pass through spaces in the bone substance, which are invisible to the naked eye. The periosteum also contains nerves which enter the bone It appears impossible that bones should have nerves, but this is a mistake, as anyone knows who has had experience of diseased bone, and the pain and aching which accompanies it, which is dull, heavy, and almost intolerable, when severe. We are only aware of our bones and their nerves when they become diseased. When the canals enclosing the nerves become inflamed and swollen, the latter are compressed, and there is pain.

Bones are not at first what they become in an adult person. In the unborn infant the parts which are to become bone are mapped out in cartilage, which is gradually converted into bone. It is a curious fact that each tissue can abstract from the blood as it traverses it what is required for its nourishment. Thus bone derives material to form itself. Muscle takes what it needs, and so on with each. No mistake is ever made, and bony matter is never found in the nerves for instance. Hence the cartilage in the unborn infant takes from the blood what is required to form bone, leaving deposits of boney material along the course of the blood vessels, and the appearance of the cartilage changes and appears granular. Further supplies of material are added to these deposits, which spread until they meet, and so the cartilage finally becomes bone.

The earthy matter is not laid down roughly and promiscuously, but so as to form a system of little plates so arranged to give the greatest possible strength in the direction where it is most needed, and to form also a network of tubes and spaces for the supply of nourishment to the bone, the whole forming one of the most wonderful and beautiful structures met with in the human body.

The change of cartilage into bone does not occur promiscuously throughout the whole mass, but proceeds from certain points in a regular and orderly manner. For instance, in the thigh bone very early in its history a point of true bone appears in the middle of the shaft, and gradually extends upwards and downwards until the whole of the shaft has become changed into bone. Then just before birth a second centre appears at the lower end and ossification proceeds from this also. At the end of the first year a third centre shows itself in the head of the bone, and in the fourth year we find another centre at the top of the bone, and lastly a fifth not far from this. These separate growing points unite with the main shaft in the opposite order in which they appeared, the last deposited joins first, and so on.

Thus at the age of 21 all the bones in the body are complete. This peculiar method of ossification has two advantages; first, it facilitates growth, because the great density of bone makes its rapid increase difficult, and hence growth takes place in the cartilage at the end of each shaft, which grows whilst being changed on each side into bone. If anything interferes with this process, so that the separate pieces of bone become joined together before their time, there is an end to all growth, and the child is either short of stature or a dwarf, according to the degree of arrest in the growth. Another advantage of this method is that during childhood and youth there is at the end of each bone a piece of cartilage which, by its elasticity, acts as a buffer in breaking shocks, and young people can jump and tumble without hurting themselves. There is one very important difference between the structure of the bones of infants and young children and those of adults.

In the long bones of the latter, as we have already said, there is a hollow in the centre filled with marrow, but in infants and young children this hollow does not exist.

The long bone of an infant is so small that were it hollow it would be quite too weak for any practical purposes, and hence Nature makes the bone solid in the first instance, and only as it grows in outward girth and size is the interior re-absorbed and made hollow.

Let us now consider the joints, muscles, and tendons, without which bones would be of little use to us, as we should never be able to move them. First as regards a joint. It is the union of two bones, which are so arranged as to allow of a great deal of movement, and consequently at one and the same time. There is union and separation of the bones which appear contradictory. There must be two bones for a joint, and there can only be movement where there is a joint. Sometimes in certain diseases, as for instance in severe rheumatism of the knee, the joint does not act, and the leg is very stiff.

In the human body we find three kinds of joints: (1) The ball and socket joint, as, for instance, where the upper arm fits into the shoulder, and the upper part of the leg fits into the thigh. Here one end of the bone is round, almost like a ball, and the other bone has a deep cup into which it fits, but can move in all directions in the cup; in fact, this joint gives us most variety of movement. The next kind is (2) the hinge joint, which only allows backwards and forwards movement, as a door moves. This kind of joint is found at the elbow and knee. Apparently we can move the lower part of the arm round like the upper, but this is due to the ball and socket joint at the shoulder, not to the elbow joint, and if the ball and socket joint at the shoulder became stiffened we could not move the lower part of the arm round. Or if the upper part of the arm be grasped whilst the lower part be turned round, the whole arm will be felt to move. The joint which move the hand and enable it to turn round is an example of the third kind. The (3) sliding joint, in which the bones slide on one another. The bones of the spine act similarly, and are, perhaps, a better example of this kind of joint. The hand is able to move in various ways, and has a wonderful variety of movement, and hence the delicacy of touch which you find in the human hand.

### Appointments.

[It is requested that all books intended to be noticed in this column may be sent to the Editor, at the Office, 428, Strand, W.C., not later than Tuesday evening in each week.]

Border Counties Home for Incurables.—Miss Kate Murphy, who was trained at Glasgow Royal Infirmary, has been appointed Matron at this institution. We wish her all success in her new work.

Harrogate Sanatorium.—Miss Fanny Taylor has been appointed Matron of this sanatorium. She was trained at Gravesend Hospital, and afterwards did private nursing in Norwich and Bournemouth. We congratulate her on her appointment.

Stockton-on-Tees Fever Hospital.—Miss Annie Bellamy has been appointed Matron of this hospital. She was trained at Westminster Hospital, and held the post of nurse at Hull Sanatorium, and Housekeeper at Fitzroy House, London.

### Character in the Insane.

By an Experienced Correspondent.

Those who work for any length of time among the insane, if only they are gifted with the seeing eye and the hearing ear, have unrivalled opportunities of studying character. Insanity, as a rule, knows no reticence; and the insane betray many points of character, which were so carefully concealed, as long as reason and judgment held the guiding rein, that they were quite unsuspected by others, and perhaps even by themselves. This is the reason why it so often seems as if brain disease completely altered the character, making a man the very opposite of all that he has hitherto been or appeared to be. It is not really so. All his qualities, both bad and good, are so much exaggerated when self-restraint is broken down, that his bad traits seem abnormally wicked, and even his good ones become ridiculous, annoying, and sometimes mischievous and hurtful.

Insanity does not create in us tendencies that have never existed, but it exaggerates enormously those that exist, and develops others that are latent. Tendencies that have been constantly checked and corrected by training and education, are yet not eradicated, though we may flatter ourselves that they are so, and reason being unseated, they suddenly come to light. Thus the insane openly show, without any sense of shame, qualities and characteristics which, as long as they were sane, reason, common sense, education, or religious principle, kept in check or completely hidden, but which, nevertheless, have always belonged to their nature, whether they themselves were conscious of it or not. The old proverb that says, "When the wine is in the wit is out," may equally apply to a brain weakened by disease.

If it is deeply interesting to study character among the insane, it is also at times both saddening and humiliating. We often look with indulgence upon what we call harmless vanity, or a natural love of admiration, in a rather frivolous girl; but look at the same girl when, by some accident or misfortune, the mental balance is overthrown. Now she believes, and does not hesitate to say, that she is perfectly beautiful, has an exquisite figure, is in every way charming and attractive, and that every man who sees her immediately falls violently in love with her. This is no exaggerated picture, but one that came under my own observation; the patient had evidently been always a rather empty-headed girl, thinking of little besides dress and flirtation, and being a victim to hereditary insanity, the result was as I have stated. It is only an absurdly exaggerated sense of self-importance that produces the very common delusion that the patient is a king or a queen, or even a deity.

Again, a natural humility and a tendency to self-depreciation are frequently exaggerated by disease into the delusion that the patient is an object of dislike and contempt to everyone, that he has committed some unnameable crime, and that consequently he is beyond redemption, and is regarded with horror by all around him. If he is of a religious turn of mind, he believes himself to be eternally lost, and sinks into a state of chronic melancholy and apathy. On the other hand, a natural self-reliance, no longer controlled by common sense, expands into a belief that the patient has done, and can do, feats beyond the power of any mortal man.

A person of an anxious and worrying disposition is a most trying patient to deal with, when the condition becomes morbid and chronic. He is always anticipating calamities. He imagines that he is totally ruined, and about to be forcibly conveyed to the workhouse, or imprisoned for debt. Some misfortune has overtaken his family, his children have been murdered, his house has been burnt down. He lives in a constant state of terror and anxiety, which is pitiable to witness and hopeless to contend with. No arguments, nor even the most irrefragable proofs, will convince him for more than a few minutes. Reasoned out of one calamity, he immediately invents another, and presently returns to the former one with all the persistence of a diseased brain. To see a patient in this distressing condition, should be a salutary lesson to those who are in the habit of meeting troubles half way, and are too apt to let their fears and anxieties outrun their common sense and reason.

A constitutionally indolent person becomes, when insane, a monument of laziness, which must be seen to be believed in. Such a patient will, if unrestrained, lie in bed for weeks together without ever changing his clothes, or washing himself, too lazy even, sometimes, to feed himself. If forced to get up and dress himself, he will sit or lie about, listless and unoccupied, not because his mental powers are altogether destroyed, but because he is without energy to use them, or even to wish to use them. A good deal of this native indolence may be observed among the coloured races, where patients will lie all day long in the sun, half asleep, not troubling to come to their meals or even to their beds, until forced to do so. But with some this indolence cannot be called a sympton of insanity, being a normal, and not a morbid condition of mind and body.

Everyone who has suffered from a good old-fashioned fit of home sickness, when many miles from home, can quite believe that the emotion might develop into insanity in a home-loving and domestic disposition. In short, we all have a mental bias of some kind; and we are all aware that the predominating tendency would run away with us, if unchecked by reason. There is the weak point which requires to be most strongly fortified; a habit of self-control counts for something, even in disease; and if we know, as we ought to know, our weak points, and are ashamed to show them, let us not be satisfied with concealing, let us continually endeavour to strengthen them; even if insanity be our fate, no such efforts will be thrown away.

Yet with all this variety of character, there is also much monotony, on the surface at least. As human nature, in its normal condition, has many points of resemblance in every individual, so it is with the insane. For instance, almost all insane persons are more or less discontented, and more or less wrapped up in themselves. They harp continually on one or two trivial themes, and are quite lost to outside interests. If they have delusions, they talk of nothing else; if they are free from delusions, they talk about their petty personal interests. It is rare to meet with a really intellectual lunatic who still retains an interest in his intellectual pursuits.

Each form of insanity has its peculiar characteristics, which are the same in every individual, however widely their dispositions may otherwise differ. The patient is either silent and apathetic, or unduly excited, he is either inordinately careful of his personal appearance, or utterly neglectful of it. Either he lacks all interest in those around him, or he is interfering and mischievous to the last degree. And apropos of this last characteristic. I have remarked that nothing is more grotesquely exaggerated by insanity, than a managing, domineering disposition. One such patient can upset a whole ward, continually annoying the other patients, the nurses and the doctors by his insane passion for setting everything right (or what he considers right), and for interfering in everyone's affairs. We greatly admire a genius for organisation in a sane person; but met with in an asylum, it is nothing short of a tormenting fiend. A person of restless energy is somewhat wearing in the outside world; what is he, then, when the scope of his activities is limited to the four walls of an asylum?

It is the monotony of lunacy, more than anything else, that makes attendance on the insane so trying to the nerves. The constant repetition of the same words and phrases, the daily conflict with bad habits which are never corrected, with apathy which is roused only to relapse immediately, with unreasoning obstinacy which cannot be reasoned with—all this produces such weariness at times, such a feeling of sameness, that the study of individual character seems hardly worth while.

And yet there is no other way but by patient and constant study of the idiosyncrasies of each patient who comes under our care to arrive at any useful understanding of what insanity really is, and of what means will be most apt to soothe and relieve, or permanently to cure it.

### On Private Nursing in Connection with Hospitals.

The relations of private nursing with the hospital and the resultant effects of the business upon the work of the hospital have not, as far at least as I am aware, received as much consideration as the importance of the matter demands, and the following views which have been forced upon me in a recent investigation of the subject may serve to throw some light upon a question which is full of difficulties, and is open to more than one answer.

It would certainly seem that hospital authorities, in at least some instances, have come to the conclusion that nothing but advantage is likely to ensue from the establishment of a private nursing business in connection with a hospital, and they have rushed impetuously into the work, influenced chiefly by the belief that large profits may be made from the concern.

The promise of profits ought, however, not to outweigh other considerations unless it is distinctly shown that the general results are advantageous to the work of the hospital; and it will be prudent to weigh advantages and disadvantages which appear likely to follow the working of a private nursing business in connection with a hospital.

There are two ways in which private nursing may be worked by a hospital: the business may be engrafted on to the hospital so that the concern is contained within the hospital, the nurses being taken from the staff nurses, housed and fed in the hospital, and the accounts considered as part and parcel with those of the parent institution; or the business may be conducted as an entirely distinct concern, the private nurses forming a body by themselves, being housed and fed in a separate building, and the accounts being kept apart from the accounts of the hospital. The second of these is wholly unobjectionable.

To the first plan many serious objections may be raised; and the advantages appear to me so inconsiderable in proportion to the disadvantages that no hospital ought, in my opinion, to carry on a private nursing business on these lines. The chief objections are the following:— The constant changes that take place in the hospital staff in consequence of the nurses being sent off to private cases are extremely detrimental to the interests of the patients in hospital. Nurses who have got accustomed to the patients and know the needs of the cases, are replaced by others who have to learn the wants of the patients and the nature of the cases; the sisters have to instruct the nurses anew, and the general work of the gallery and the wards is subject to incessant change which much interferes with the regular work of the hospital. The physician or surgeon in charge feels that the nurses do not understand the cases, while the patients resent few things more than a change of nurse.

It is also very unsatisfactory as regards the nurses, for a nurse may be sent away at any time to an urgent case; she may be summoned at a time when she ought to be resting or having her meals, and she may be even called up at night when she is tired to death with the day's work in the wards. It has also a bad effect upon the general condition of the staff nurses, inasmuch as the change, the opportunities and the excitement of private nursing may tend to make hospital work distasteful and wearisome, and the private nurse is apt to return to staff work discontented and unsettled. Moreover, there is an inclination to laxity in discipline, the private nurse having matters pretty nearly in her own hands, and in most instances having had much more time at her disposal that she is likely to have in hospital.

These appear to me to be very cogent reasons against running a private nursing concern directly from a hospital.

As to the arguments sometimes adduced in favour of the plan, they amount in my opinion to very little.

The advertisement of the hospital by such means is not a very bold, or potent advertisement, and as to the probability of legacies being left to the hospital in grateful remembrance of the services of a particular nurse, that can hardly be a very frequent occurrence; so that as far as I can judge, the arguments in favour of the practice of working the private nursing from the hospital are few and feeble.

When a private nursing institute is kept entirely distinct from the hospital the same objections do not apply; and if the business is mainly conducted by a home sister, and the nurses kept away from visiting the hospital, it may be admtited that the institution will be a convenience to some of the medical profession and the public who employ the nurses; but certainly as regards benefit to the hospital, the good done hardly appears to be commensurate with the trouble, unless the private nursing institute be looked upon as a reserve upon which the hospital authorities may draw, if from any pressure, or from the absence of nurses at holiday time, there is any deficiency in the number of the working staff. In this way it becomes useful to the parent institution, and a mean whereby well-trained and trustworthy nurses may be attached to the hospital for future necessities. R. E. T.

### Workhouse Infirmary.

The sanitary condition of Hatfield Workhouse Infirmary, and the structural and nursing arrangements there, formed the subject of the second article in the *British Medical Journal*, on "Nursing and Admi nistration of Provincial Workhouses and Infirmaries." The heating and ventilation of the wards appear to need re-modelling, and the maternity attic is described as most inadequate and unsuited for a lying-in ward. It is however probable that the guardians will see the advisibility of speedily reforming these things. Stress is laid on the exceeding dreariness of the airing grounds of the patients, and of the flower garden being equally out of their sight and reach. The third article, which appeared last week, is devoted to the St. Albans Workhouse. It is in contemplation to supersede the present inadequate old buildings by new ones. One trained nurse has charge of the fifty-eight beds, and she has an assistant who has had "some" experience. Of night nursing there is none, except when an urgent case necessitates the presence of the day nurse. At other times the wardsman or woman "sleeps in the wards." The *British Medical Journal* calls special attention to this practice, which, by the way, is one that has often been severely criticised by us.

### Wants and Workers.

Could anyone tell me of an establishment near Birkenhead or Liverpool where nurses are taken in for quarantine after infectious cases?—*Quarantine.*

Will any nurse having Gounod's "Nazareth" kindly oblige me by copying out "Though Poor be the Chamber," &c., and sending them to me at the office of this paper addressed to *Sister Winifred*?

A correspondent would be glad to hear of nurses or others with a sufficient knowledge of Dutch for translating purposes. Address the Editor of The Hospital, 428, Strand, W.C.

An anxious father will be glad to know of a suitable home for his son of twenty-four. Although unfit for ordinary occupation by a paralysed left arm, there are many things he can do. A moderate sum would be paid to an institution where he would be kindly treated and made useful.

### The Children's Country Holidays Fund.

A PRIVATE PATIENT.

"My little girl seems out of sorts, doctor, and she has lost her appetite," says the wealthy mother. "What do you advise?" After a brief examination the West-end physician replies: "Let her have a change and run wild in the country for a month, and don't let her be worried with lessons." And the pleasant prescription is carried out in its entirety with the happiest results. Health of mind and body come back to the languid child, and she returns to her school books with renewed vigour and increased intelligence.

AN OUT-PATIENT.

"Can't you take the boy in, please, sir?" says an anxious-eyed mother. "He's not fit to go to school. His teacher says he isn't doin' a mite o' good in 'is class; but they don't punish 'im, 'cos they says they can see as 'e ain't well, poor little chap." The doctor shakes his head: "I can't take him into the ward, my good woman; it wouldn't do him any good if I did. You see, he isn't ill; he's just run down, and he wants to pick up again, but its *country* not *ward* air that he wants. Haven't you any friends in the country who would have him for a month and feed him up a bit? He only needs fresh air." "I haven't got any friends out o' London," says the woman despondingly. "I guess he'll just fade away like my other little 'uns," and the poor woman fairly breaks down, unaware that the harassed physician is still devoting a few valuable minutes to her case. He has a general idea that the Children's Country Holiday Fund might serve her turn, and so he is hastily writing down the address to hand to her.

AN ANNUAL OUTING.

Everybody wants a periodical holiday, but, unfortunately, a great many people never get one. The weary mothers, even if they had a chance, "free of expense," could not muster up decent clothes to go away in. But the children can go, and they ought to go, every one of them.

The sound ones because they need to be kept sound, and the sickly ones because they require every possible assistance in becoming healthy.

The days are, happily, over when "poor health" was considered an unalienable possession, and the feeble child was expected, as a matter of course, to become a feeble man; modern knowledge having proved that intelligent care and hygienic surroundings can exterminate many youthful weaknesses. But with increase of knowledge has come enlarged responsibilities, and who can remain coldly indifferent to the claims of the little ones?

As to the quality and quantity of "free education" bestowed on the children of to-day opinions may naturally vary. But there can be no division on the vital question of health.

A PRIVILEGE WITHIN REACH OF EVERYONE.

To build up the weakly, and to strengthen ailing children, has become a privilege in which all can share. If every prosperous man or woman who plans a seaside or coun ry sojourn for his own carefully-cherished children, would include in this annual outlay a subscription to a fund for poor children, there would be no further need of urgent appeals from pulpit or platform. A meeting in aid of the Children's Country Holiday Fund was held at the Mansion House on the 15th inst., the Lord Mayor presiding. It was computed that subscriptions to the amount of £60 had been received in consequence of the Lady Mayoress's invitation. In the course of last year 28,589 children enjoyed holidays, but the subscriptions, including the parents' payments, fell considerably short of the expenditure. It is to be hoped that each year an increased value will be set on the moral and physical results of Children's Country Holidays, and then subscriptions will pour into 20, Buckingham Street, Strand, where particulars of the fund can be obtained from Mr. Cyril Jackson. Surely all who realise what an annual visit to the country must mean to the dwellers in squalid houses in unlovely streets, amidst unsanitary surroundings, will cordially respond to the eloquent appeal so kindly made last week at the Mansion House by the Lord Mayor, Lord Knutsford, Archdeacon Sinclair, and Mr. W. S. W. Smith, M.P.

### Nursing in Germany.

BY A CORRESPONDENT.

A NEW PROPOSED HOSPITAL.

THE House Insurance Company in Lübeck has decided to erect a hospital for persons suffering from diseases of the lungs out of the surplus funds devoted to the benefit of those who belong to their company. It is to be built in the Harz Mountains, and will contain accommodation for one hundred patients. The accomplishment of this project will be a step of great importance, and it is to be hoped the good example set by Lübeck will speedily be followed by other insurance companies.

The Grand Duke of Saxe-Weimar has conferred the Cross of Merit of the Order of the Falcon upon Sister Margarete Lene, working at the Station Cameroon in connection with the Women's Nursing Association in the Colonies, in recognition of the valuable service rendered by her during the revolt in December, 1893.

The construction of a portable and most economical water-bed for military use is proposed by a military surgeon, Dr. Kirchuer, of Osuabrück. As cases giving rise to this necessity rarely occur in garrison the expense of an india-rubber bed, such as is in use in civil hospitals, would be too great, and he suggests strong waterproof sail-cloth or canvas as a substitute, supported by a frame-work of wood or iron, which could be easily taken to pieces, and be of use in time of war. To avoid the heavy pressure of water, iron feet at the bottom could be constructed, and would serve to preserve the full width of the bed. The water could be removed daily by means of a plug, and the interior of the bed could be easily and thoroughly cleansed by hot water and soft soap after the removal of the water. A case of acute muscular rheumatism which came under Dr. Kirchuer's notice, where much inconvenience and unnecessary suffering was caused through want of a water-bed, has induced him to give utterance to his ideas on the subject.

Dr. O. Ammann, of Munich, is the inventor of a very convenient and inexpensive appliance to simplify the bathing of infants. It consists of a kind of net, composed of a texture which will not, of course, hurt the child, supported on each side by three girths, one passing under the knees, one under the back of the neck, and the third to ensure perfect security and support to the head. The girths can be heightened or lowered according to the quantity of water the bath contains, and are fastened by means of hooks, which can be affixed to any kind of bath. The net is made to exactly fit the form of a child lying in a normal position. The feet only are left unprotected; in this position the head, ears, eyes, feet, and hands especially can be thoroughly and easily washed. The main advantages of this simple contrivance are that no accident, such as drowning, sprains, &c., can possibly occur to the child lying in the net in the water, the mother or nurse has both hands free, and spares herself the fatigue of supporting the child, and should some article be orgotten, as is unfortunately often the case, the nurse can-safely leave her charge and fetch it, whereas it seems now as if the presence of two persons were necessary at the morning ablutions of the youngest of the family. A larger size net can be obtained for older children's use in case of illness.

The last number of the *Zeitschrift für Kraukenpflege* contains a long and able article on "The Treatment and Nursing of Small-pox Patients," by Dr. L. Ppeiffer, of Weimar, consulting physician; also a lecture on "The Preparation of Medicinal Baths," delivered by Dr. W. H. Gilbert, of Baden-Baden. Dr. Emil Guttmann, of Leipzig, is the author of a practical and comprehensive book of instruction to hospital and home nurses.

### For Reading to the Sick.

### CHRIST'S SYMPATHY.

**Motto.**

'Tis, His heart that's touched with all our joys,
And feeleth for our grief. —*Faber.*

**Verses.**

How was He,
The Blessed One, made perfect? Why, by grief—
The fellowship of voluntary grief—
He read the tear-stained book of poor men's souls,
As I must learn to read it. —*Kingsley.*

Thou know'st our bitterness! Our joys are Thine!
No stranger Thou to all our wanderings wild!
Nor could we bear to think how every line
Of us—Thy darkened likeness and defiled—
Stands in full sunshine of Thy piercing eye,
But that Thou call'st us Brethren! Sweet repose
Is in that word; the Lord who dwells on high
Knows all, yet loves us better than he knows.—*Keble.*

**Reading.**

Our Lord wept over Jerusalem because of its sin. . . . He loved that fair city, but, above all, He loved the souls of men, and He wept because He felt sorry for the miseries which befel them, whether from their own fault or not. The kind and tender sympathy of our Lord with the sorrows of men makes His human character one of surpassing loveliness, a character which has had a calm and soothing influence upon the world's passions, and which has said to the raging waves, "Peace! be still!" And how many weary hearts have been comforted by turning from the coldness, the self-will, "the don't care for you" of the children of men, to the kindness and love of the Son of God? . . . Our Lord's sympathy is as active now as it was when He was on earth. In all our affliction He is afflicted, even in His home of Glory. What should we do without that sympathy? Our sorrows would be overwhelming without it, our hearts would be broken and crushed without the sympathy of Jesus, the living sympathy, living now as active and as true as ever. . . . But not only have the faithful disciples of Christ ever since He lived and died been better for His sympathy, but the world itself has been better for it. Those who have little or no title to be called Christians have cultivated the better impulses of their nature, and have been more active in their kindness. . . . The world, however, is not the place where the manifestations of our Savour's sympathy are chiefly to be seen, but in the church, amongst His own sheep, who hear His voice, and are known to Him by name. To them He is an ever-present helper.

Well I know thy trouble, O my servant true,
Thou art very weary, I was weary too.

"Thou art very weary." Here is the disease. "I was weary too." Here is the cure. It is enough for the servant to be as His master, because he knows that the Master feels for him. If all human sympathy be denied us, as it was denied to the poor and holy Lazarus, who had no comforters but the dogs wandering about the streets, we may have the Saviour's sympathy if we will. He is standing ready at the door. "Behold," He says, "I stand at the door, and knock; if any man hear My voice, and open the door, I will come in unto him and sup with him, and he with Me.—*Rev. R. Adams.*

Here is that which gives its reality to the life of redeemed men; here is that which fills and glorifies every earthly relation—that Christ the true man Himself partook of them; that He took on Him and surrounded Himself with the bonds of family life; that He with whom all was reality, was what we are now; that in Him, therefore, and through Him, that life which without Him was an empty and deceiving shadow, grows into a great reality; that in bearing our very nature, He hath for every faithful man for ever raised and glorified his new and ransomed life in all its parts. For He has showed us that we may as men, and in the things of men, truly serve the Lord our God. —*Bishop Wilberforce.*

### Notes from Melbourne.

(Communicated.)

There is a war in the Presbyterian Assembly and in our newspapers anent the splendid work Mrs. Armour (now dead) and Miss Sutherland have been doing for years past among our Melbourne waifs. Mrs. Armour gave home, money, and time to the care of neglected children. Miss Sutherland with untiring energy picked them out of the gutters and found good homes for them in the country away often from drunken and utterly wretched parents and surroundings; but a difficulty has arisen between the workers, and it seems, according to the Press, to rest somewhat on sectarian grounds, which it is to be hoped will not, however, be allowed to swamp the usefulness of the work.

The consumptive wing at the Austin Hospital was partially reopened on Thursday, April 26th, by the Mayor of Melbourne in the presence of a large party of visitors, and every bed was soon filled, the new patients, many of whom appeared very ill, expressing great thankfulness for their admittance to the fine wards of their beautifully situated hospital. The Hon. Treasurer is making urgent appeals through the Victoria Press for funds to enable the committee to carry on the hospital without allowing it to be overtaken by debt.

Our hospitals in town and country get very crowded, especially at this time of year (autumn); the aged come in for shelter and help, there being no place else for them to go to, unless, indeed, as sometimes happens, they are sent to gaol simply to be taken care of. Port Melbourne, a city suburb, is backward in sanitary matters. Typhoid continues in country places; it is now in Romsey, a place low-lying, with a long reputation for attacks of typhoid.

### An Emergency.

By A Nurse.

A man of thirty-eight, and a little child of three years old, having been badly injured in a burning house, were carried into a room two doors off, by the firemen. I happened to pass by in nursing dress, and was called it. I dispatched someone in the crowd for a doctor and another for oil, and a third for brandy, &c., then hurried into the room and quickly cleared it of all persons except two women. The man was just regaining consciousness, and the little child crying piteously. The latter was soon placed in a warm bath, the temperature of which I tested with my elbow; she was badly burnt about her legs and lower part of her body. The bath soothed and quieted her almost directly, and she cheerfully and gratefully drank some warm milk which one of the women had prepared by my direction.

In the meantime the man was more seriously hurt. Having loosened the things round his neck, I ripped his sleeve and trousers up and carefully removed them from his injured side. In some places it was necessary to pour a little oil on the clothes as they had stuck to the wound. I then covered the injured parts as quickly as possible with old linen steeped in linseed oil, so as to exclude the air. During the time I was doing that, the woman who had warmed the milk persuaded him to drink about ℥iv. of it with ℥ss. of brandy added.

Great thanks are due to the two women, who did the tasks I set them quietly and quickly. They did not ask any useless questions, but obeyed implicitly in everything, just like well-trained probationers!

I was in the middle of washing the man's face—as it was very smoky and dirty—when the doctor arrived, and he thanked me warmly for the assistance I had rendered.

Duty calling me elsewhere I was obliged most regretfully to depart, but I heard from time to time that both patients were getting on well, and they eventually recovered their former health, but unfortunately they are terribly disfigured.

☞ For The Muses' Looking Glass, Everybody's Opinion, Book World for Women and Nurses, &c., see page cxix. et seq.

### The Muses' Looking Glass.

### THREE EMPRESSES.*

THIS latest work by Miss Gearey has just passed into a second edition, and one cannot be surprised that it is so, because, besides being of a certain historical value, a romance of no mean order pervades the pages of her book. For more romantic lives have not been written than those of Josephine, Marie Louise, and Eugénie, the three luckless Empresses of France, whose biographer Miss Gearey at present constitutes herself.

The irony of fate has, perhaps, never been more definitely brought before our minds than in the instance of the ephemeral sway of these unfortunate women. Of the three lives here contrasted together, our sympathies are most drawn to the one with whose story the authoress commences her narratives, with the lovely Empress Josephine, that is. It was in a patriarchal but simple household in Martinique that we are first introduced to the woman who, in after years, graced the commanding position of leader of society, beauty, and fashion in Europe. Martinique, where she was born, is an island which has been spoken of as the Pearl of the Antilles. Nurtured amidst tropical vegitation, in a land of orange and lemon groves, little wonder is it that the grace and charm, the poetical imagination of the Creole combined to render the exquisite beauty of Mademoiselle de la Pagerie of an extraordinarily attractive order.

Destined in marriage to give her hand to a certain young officer of noble family called Beauharnais, at an early age the girl quitted Martinique, accompanied by her father. It is reported that upon the eve of her journey she consulted an ancient negro woman, far-famed for the power of her prophecies. What she read in the face of her fair consultant was, "a share of happiness, and an equal share of unhappiness." "You will marry very soon," added the negress, "but your union will not be a happy one; you will become a widow, and one day you will be Queen of France, and something more than a Queen, but it will not be for long!"

How unlikely it seemed at first that the visionary spoke the truth was apparent to none more than to the young beauty herself. Queen of France—and something more, too—a fate which in days to come proved only too real for Mademoiselle Josephine de la Pagerie!

The very year she landed in France, there landed, too, a boy of ten years old—sent from the Island of Corsica to pursue his studies at the Military College of Brienne. A boy who, later, was destined to hold Europe under his sway, as Napolean, crowned Emperor of the French. Josephine's life, upon her marriage with the young Viscount de Beauharnais, was not a happy one. Two children were born of this union—the Prince Eugene, and Hortense, afterwards Queen of Holland, and mother of Napoleon III. Not alone domestically was the Viscountess's existence unhappy, but surroundings combined to make her one of a group of helpless, terrified women who stood trembling and clinging to each other in horror and amazement at the awful crimes perpetrated around them at this time in the sacred name of Liberty. Madame de Beauharnais subsequently became one of many who were thrown into prison, though she was spared from joining the ranks of those who left the gloomy walls only to know death through the cruel and repulsive instrumentality of the guillotine.

Calmer days succeeded. France was governed by the Directory. Paris breathed again. And the Parisians, irrepressible as is their nature, forgot the horrors of the past in the wild extravagances of the luxury and splendour of the present. At this time it is that Josephine, still young and in the zenith of her beauty, is thrown with the youthful Napoleon. Bonaparte was one of the many young men, writes one of his more contemporary biographers, who were much struck by the fascinations and the graceful beauty of Madame Beauharnais, who was greatly superior to the rest of the circle in which she moved, both by reason of the name she bore, and from the elegance of her manners. But Josephine's friends were much astonished to learn subsequently that the widow of M. de Beauharnais had promised her hand in marriage to a man so little known as Bonaparte! And yet all Europe was destined to bow later on to the meteor-like achievements of this remarkable soldier. Full of indomitable ambition as he was, yet his love for the gentle object of his affections was of an equally strong character. Miss Gearey gives us many of the letters which passed between him and his bride after their marriage, and when he left her side to prosecute his earlier "victories of destiny," as he named them.

One specimen of these letters we now extract, which illustrates something of the inner workings of the man's life, whose public side has ever been better known than his private one. "Soul of my soul," it runs, "write to me by every courier, otherwise I cannot live. I am very occupied here (Albenga). Beaulieu moves his army; we are in sight; I am a little fatigued. Adieu, adieu, adieu. I am going to sleep. Sleep consoles me; it places me by thy side. But, alas, on awakening I find myself 300 leagues from thee."

And later, "I am not pleased," he writes. "Your last letter is as cold as friendship. I have not found in it the fire which kindles your looks, and which I believe that I formerly saw. But that is my caprice. I found that your first letters oppressed my soul too much; the revolution that they produced attacked my rest and enslaved my senses."

The wife's letters, unfortunately, are not preserved; but as has been pointed out, the exigences of love did not induce the soldier to neglect war, for Napoleon continued to follow up with extraordinary precision the successes which he ha already attained. And Josephine shared in his glory. On the conqueror's return to Paris, the fêtes and gaiety which were daily and nightly held read like a fairy story. But, alas, the proud and beautiful Josephine was not destined long to enjoy the dazzling brilliance of such a life. Her fortunes turned at the death of her son; the funeral dirge of the young Prince rang, too, the death-knell of a woman's hopes of happiness in this world. Every word of Miss Gearey's account of this sorrowful time are well worth reading, and a more than ordinary degree of interest attaches itself to the unmaking, as to the making of the Empress.

A little later on, as we all know, the Imperial decree went forth that another consort was to replace the childless Empress, and the brilliancy of the French Court of the Tuilleries was to be changed for the vanquished woman by the quiet asylum offered her in one of the Imperial country seats. "To quit the throne," the bereaved woman remarked, "is nothing to me . . . but to love at the same time the man to whom I have consecrated my dearest love, that sacrifice is beyond my strength."

We have described at some length the life of the first of Miss Gearey's Empresses, and space denies much mention of the other two, Marie Louise and Eugénie, both of which lives form almost equally interesting themes as the Empress Josephine's. We cordially commend the perusal of this delightful book to all the reading public, a work which will be found of so absorbing an interest that it is hard to tear oneself from its pages and return to the less eventful existence of our own more ordinary surroundings.

* "Three Empresses." By CAROLINE GEAREY. (London: Digby, Long, and Co.)

### Everybody's Opinion.

[Correspondence on all subjects is invited, but we cannot in any way be responsible for the opinions expressed by our correspondents. No communications can be entertained if the name and address of the correspondent is not given, or unless one side of the paper only be written on.]

DISTRICT NURSING.

"The Matron of a District Nurses' Home" writes: I have read with much surprise Miss Mary Mason's opinion of the qualifications that she considers necessary for district nursing. Before answering her objections to fully-qualified nurses undertaking this work amongst the sick poor in their own homes, I should state that I have been for nearly five years district nursing, both as nurse and matron, in which latter capacity I am at present acting, having five fully-qualified nurses with me. I feel, therefore, that I can speak with more authority than one who, from her letter, has had a very limited experience. I should like, if you could possibly afford the space in your valuable and widely-read paper, to append the list of diseases which we have had to nurse during the last year in our several districts; I think it would, in itself, be proof enough to show that certainly three months' training was inadequate for those under whose care such acute suffering comes. Since Miss Mason thinks "an occasional typhoid" is about the most acute case we can have, may I mention that we had seventeen such cases on our books at once, and I think no medical man or experienced nurse would ever be satisfied or at ease leaving such cases to a three months' probationer, considering how very seldom the doctor is met by the nurse on her rounds. As to our "highly trained nurses overstocking our districts," may I say from my experience that though our association gives its nurses good salaries (but not £50 per annum), as stated by Miss Mason, I have had a vacancy on my staff for five months, and the association has had to pay an institution at the usual weekly rate for a locum. I think Miss Mason cannot be familiar with the wants of the sick poor, if she would, from her very limited experience, supply them with—not a nurse—but a woman. Perhaps had she (before taking up district nursing as her calling, or her pen to write of such) come with me from one of the largest children's hospitals in London where, as "sister," I followed the little patients who had been discharged into the slums, she would change her opinion, and I hope throw all her weight of experience into the needs of the sick poor. There is, I should say, infinitely more tact and gentle womanliness required than in any hospital nursing. In fact, any woman almost can be "broken in," if need be, to hospital work, where the constant attendance of doctors and sisters will see any negligence on the part of the nurse, whereas in the district a nurse must stand on her own showing. No nurse is fit for such responsibility whose work needs supervision. Miss Mason is evidently not cognisant with the organisation of these "large societies" which have the care of the sick poor, or else she would know that the nursing is far from being "very precarious." She would do well to hear the opinions of the doctors and patients as to the prompt and efficient way their cases are taken up, either chronic or otherwise. Who but Miss Mason (calling herself a nurse) would give phthisis and paralysis unskilled nursing?

KIMBERLEY HOSPITAL.

The Bishop of Bloemfontein writes: My attention has been called to a letter in the "Nursing Supplement" of your issue of June 2nd, in which a violent attack is made on the nursing system at the Kimberly Hospital by a gentleman who recently held the position of house surgeon there for a short time. I think there are very few persons in Kimberly or out of it who would agree with Dr. Bishop in his assertion that the interests of the nurses there are not "protected by a just and impartial board." And I would suggest to any persons interested that, before accepting his assertions as representing the true state of the case, they should make further inquiries, say, of the Mayor of Kimberley (James Lawrence) the Civil Commissioner and Resident Magistrate (E. A. Judge), or any other of the leading residents, medical or lay. I am confident that their testimony would be emphatic to the efficiency and excellent management of the hospital, and in particular of its nursing staff.

HOLIDAY HOMES

E. M. T. writes: Will some one kindly give me some information regarding "Holiday Homes for Nurses" at Margate, or at a seaside resort within easy distance from London? A home where no rules are attached would be preferred.

SUMMER HOLIDAYS.

"District Nurse" writes: Living alone in a beautiful part of Cheshire, with two spare bed-rooms in my house, I shall be glad to welcome any nurse unable to provide herself otherwise with a thorough change of air, and who would care to accept an offer of this kind. She must be content to wait entirely on herself. I offer a pleasant home for a couple or so of weeks, only asking a small sum to cover the necessary expenses of extra washing and cleaning—say 5s. or 7s. weekly. Return fare from Euston, third class, 29s. I have a pony trap for my long journeys, in which a visitor could always have a seat. Any further particulars may be had on applying to "Nurse Mary," 12, Teddington Park Road, Teddington, Middlesex.

### Where to Go.

A Strawberry Fete and Sale of Fancy Work will be held at Ranston on Thursday, July 5th, in aid of the Nursing Benefit Clubs of the Dorset Health Association and County Home for Training Cottage Nurses.

Invalid Children's Aid Association.—Annual meeting on Friday, June 29th, at Three p.m. To be held at 24, Park Lane, by kind permission of the Right Hon. Lord Brassey, K.C.B., who will take the chair.

Royal Albert Orphan Asylum, Bagshot.—A concert, conducted by Alberto Randegger and Mr. Fountain Meen, will be held on June 30th in aid of the funds of this institution. T.R.H. the Duke and Duchess of Connaught will be present. Tickets for the concert (10s. and 7s. 6d. each) may be obtained at the asylum, or at the offices of the institution, 62, King William Street, E.C.

Royal Institute of Painters in Water Colours.—Prince's Hall, Monday, June 25th, to Monday, July 2nd. The exhibition of pictures in aid of the funds of the Dental Hospital, Leicester Square, will be opened at three p.m. on the 25th inst. by the Duke and Duchess of Saxe-Coburg and Gotha.

### Entertainment.

An excellent entertainment in aid of the St. Elizabeth's Hospital was given last week by the Dowager Duchess of Newcastle. The entertainment consisted of a series of living pictures, tableaux, charmingly rendered by the number of ladies and gentlemen who had devoted much time and trouble on behalf of the charity.

### Notes and Queries.

### Queries.

(86) *Stewardess.*—Kindly tell me where to obtain particulars respecting nurses as stewardesses.—*Tronto.*

(87) *L.O.S.*—How can a person with some nursing experience and training gain their diploma without a lengthened absence from home?—*C. G.*

### Answers.

(86) *Stewardess* (*Tronto*).—See answer last week (81).

(87) *L.O.S.* (*C. G.*).—You had better call on the Hon. Sec. of the Midwives and Trained Nurses' Club, 12, Buckingham Street, Strand, some afternoon, and ask advice. If you write, send stamped envelope for reply.

### The Book World for Women and Nurses.

[We invite Correspondence, Criticism, Enquiries, and Notes on Books likely to interest Women and Nurses. Address, Editor, The Hospital (Nurses' Book World), 428, Strand, W.C.]

Stanhope of Chester. 1 vol. By Percy Andreæ. (London: Smith, Elder, and Co., 1894.)

Those who care about ghost stories will find a very engrossing one in "Stanhope of Chester." The ghost himself is so very substantial and uncreepy that one's interest is sustained to the end in wondering if he be a ghost at all. The leading characters are hard-headed business men, and, as such, are good subjects for commanding the confidence of the reader. The story tells of vengeance enduring beyond the grave, and gradually leading its victim on to despair and suicide. The great merit of "Stanhope of Chester" lies in the simple and apparently candid way the tale is told. The trial scene, where an innocent man is tried for murder, is quite one of the best of its kind, and, though dramatic in situation, is described with a quiet impressiveness which makes it seem very real. A good deal of connubial banter might well have been dispensed with, and does not add to the interest of the book, but the plot itself has the great merit of originality, and is capitally worked out. "Stanhope of Chester" would be an excellent book to keep one awake if obliged to sit up at night, and is not so disturbing or frightening but that one could keep on good terms with the shadows in the corners while reading it.

The Story of a Modern Woman. 1 vol. By Ella Hepworth Dixon. (London: Heinemann. 1894.)

In giving this title to her book Miss Dixon is surely a little rash, for we have all had enough of the modern woman. She has, however, treated this worn-out subject with wonderful freshness. The story is slight, and it is in the delineation of one character, that of Mary Erle, who here represents modern woman, that the interest is concentrated. The opening chapter tells of the death of Mary's father, and describes with remarkable power the house of death. The desolation of the young girl sitting in the darkened library amid the mocking scent of exotic flowers is so realistically described that this chapter alone demonstrates Miss Dixon's skill. The drawing of the male characters is rather merciless. The two chief ones are Vincent Hemming, who makes love to Mary Erle, and Dr. Dunlop Strange, who pays his attentions to Mary's great friend, but who is frustrated by the discovery of his perfidy to another woman. Mary Erle and Hemming become engaged, but one feels at once what a broken reed she has chosen to lean upon. He goes abroad, and the chapter telling of the evening of his return is one of the most powerfully written in the book. It is the old story of the woman waiting for the man who in the meantime, is making love to another woman. The story ends by Vincent Hemming, after his marriage with the other woman, coming back to ask Mary Erle to elope with him; but she refuses, though still in love with him, on the grounds that she will not do his wife so great a wrong. All through the book Miss Dixon dwells with insistence on the duty of woman to her sex. All women, however disposed to resent a novel written with a purpose, must feel that a purpose so good as that of "The Story of a Modern Woman," and one dealt with so artistically, calls for no further justification on the part of the writer.

Theophile Gautier. By Maxime du Champ. Translated by J. E. Gordon, with preface by Andrew Lang. (London: T. Fisher Unwin.)

This is the life of a great but disappointed man who, by the force of circumstances, and from want of energy and self-reliance, was doomed to leave his memory deep buried in the inaccessible files of a thousand newspapers, and in a few slender volumes of prose and verse. From a writer as prolific as Dumas, with a style as pure as that of Victor Hugo, and with the freshness of Alfred de Musset, literature had the right to expect a richer record. It was his honesty of purpose, and to the uprightness of his character that the world to-day lacks more honourable monuments of his talents. At the commencement of his career poverty and force of circumstances impressed him into the ranks of journalism, and in the fetters and shackles of a journalistic hack he ended his days. He never had the courage to throw up the permanent post of dramatic and literary critic, with its permanent but meagre pecuniary reward; he never had the courage to launch out for himself into the independent world of literature with its vast possibilities, but uncertain and speculative chances. He utterly underrated his talents, and mistrusted his powers of earning an independent livelihood. His uprightness of character forbade him to have recourse to the doubtful but certain expedients open to all journalists of amassing a goodly fortune—the sinews of a career of independent literature. His own editor thus writes of him: "Gautier," he said, "is a fool who understands nothing about journalism. I put a fortune into his hands; his work on the paper ought to have brought him in 30,000 or 40,000 francs a-year—he never knew how to turn a penny by it. There is not a theatre manager who would not have given him an income for the purpose of having him as a speaking-tube. He is at present, and since he left *La Presse*, on the *Moniteur Universal*, that is to say, the official organ of the empire. He makes nothing out of it; and I repeat, he is a fool who has never profited by his opportunities." His biography is one that well repays the reading. We are elegantly introduced to a seductive poet, an able critic, a journalist, and an honest man.

The Man in Black. By Stanley Weyman. (1 vol. London: Cassell and Co.)

This is a tale of bygone days of France when the Great King sat on the throne and Cardinal Richelieu reigned. The story opens with a picturesque description of a fair held at Fécamp, where the steep slopes and hillsides swarmed with stilt-walkers, jugglers, paste-board giants, &c. Here we find the hero of the story in a small boy walking a tight-rope. The boy had been bought by his master from some gipsies. At the fair he is again stolen, and taken into the service of the Man in Black—an astrologer and vendor of poisons. Those who rejoice in tales of dark mystery and conspiracy will delight in the account of the house of the astrologer, with its hidden room and cloaked visitors. The boy, who calls himself Jehann de Bault, eventually turns out to be the lost brother of Madame de Vidoche, who comes one night to ask the Man in Black to give her a love philtre with which to regain the love of her husband. M. de Vidoche was at hat moment with the astrologer bargaining for some poison to give to his wife. Jehann, hidden in the dark-room, overhears the conspiracy, and sees his master give the lady a love philtre which he knows to be poison. By running after her as if with a message, Jehann persuades her to give the philtre to her husband instead, as being more effective. Acting on this advice, she innocently poisons her husband, and is accused of his murder. There is a very vivid and powerful description of the scene in court. The trial is treated as one of the Christmas pastimes, and the King and Cardinal Richelieu are present. There are gruesome allusions to tortured witnesses, and the whole account is typical of the way in which justice was administered in the days of the Great King. It would be a pity to spoil the story for readers by telling the end, which comes all too soon. Suffice it to say that "The Man in Black" is a good story well told, and placed at an epoch which lends itself to horrors happily now out of date.

